# Impaired Satiation and Increased Feeding Behaviour in the Triple-Transgenic Alzheimer's Disease Mouse Model

**DOI:** 10.1371/journal.pone.0045179

**Published:** 2012-10-04

**Authors:** Adedolapo Adebakin, Jenna Bradley, Sarah Gümüsgöz, Elizabeth J. Waters, Catherine B. Lawrence

**Affiliations:** Faculty of Life Sciences, University of Manchester, Manchester, United Kingdom; Sapienza University of Rome, Italy

## Abstract

Alzheimer's disease (AD) is associated with non-cognitive symptoms such as changes in feeding behaviour that are often characterised by an increase in appetite. Increased food intake is observed in several mouse models of AD including the triple transgenic (3×TgAD) mouse, but the mechanisms underlying this hyperphagia are unknown. We therefore examined feeding behaviour in 3×TgAD mice and tested their sensitivity to exogenous and endogenous satiety factors by assessing food intake and activation of key brain regions. In the behavioural satiety sequence (BSS), 3×TgAD mice consumed more food after a fast compared to Non-Tg controls. Feeding and drinking behaviours were increased and rest decreased in 3×TgAD mice, but the overall sequence of behaviours in the BSS was maintained. Exogenous administration of the satiety factor cholecystokinin (CCK; 8–30 µg/kg, i.p.) dose-dependently reduced food intake in Non-Tg controls and increased inactive behaviour, but had no effect on food intake or behaviour in 3×TgAD mice. CCK (15 µg/kg, i.p.) increased c-Fos protein expression in the supraoptic nucleus of the hypothalamus, and the nucleus tractus solitarius (NTS) and area postrema of the brainstem to the same extent in Non-Tg and 3×TgAD mice, but less c-Fos positive cells were detected in the paraventricular hypothalamic nucleus of CCK-treated 3×TgAD compared to Non-Tg mice. In response to a fast or a period of re-feeding, there was no difference in the number of c-Fos-positive cells detected in the arcuate nucleus of the hypothalamus, NTS and area postrema of 3×TgAD compared to Non-Tg mice. The degree of c-Fos expression in the NTS was positively correlated to food intake in Non-Tg mice, however, this relationship was absent in 3×TgAD mice. These data demonstrate that 3×TgAD mice show increased feeding behaviour and insensitivity to satiation, which is possibly due to defective gut-brain signalling in response to endogenous satiety factors released by food ingestion.

## Introduction

Alzheimer's disease (AD) is a chronic progressive neurodegenerative disorder that is characterised by the accumulation of extracellular beta amyloid (Aβ) plaques and neurofibrillary tangles composed of hyperphosphorylated tau [1]. AD patients present with complex cognitive impairments, with memory loss being one of the earliest clinical symptoms. Patients with AD also suffer from several non-cognitive behavioural symptoms, including depression, anxiety, agitation, hyperactivity, disturbed circadian rhythms, alterations in eating habits and changes in energy balance such as weight loss [2]–[7]. Importantly, non-cognitive behavioural changes in AD can lead to a poorer quality of life and, in some cases, such as severe weight loss, can be life-threatening [8]–[10]. In spite of their serious consequences, the majority of non-cognitive changes in AD are not commonly studied and as such remain poorly understood. Understanding when and how these non-cognitive symptoms of AD occur could lead to a better quality of life for AD patients.

Altered eating habits are a core symptom of AD and are commonly found at some stage of the disease. Changes in eating behaviour reported in AD patients include both increased and decreased food intake [11]. AD patients also show a change in preference for the type of food consumed and a shift in circadian patterns of food intake [11]–[16]. Up to a third of AD patients present with hyperphagia and eat increased quantities of food [11], [17]–[24]. Hyperphagia has also been recently reported in several mouse models of AD including 3×TgAD mice, which present with both Aβ plaques and neurofibrillary tanges [25] and other AD mouse models, which develop amyloid deposition only [26], [27].

The mechanisms underlying hyperphagia in AD are unknown, but may be due to a reduced responsiveness to satiety factors. Satiety is the process that terminates the desire to eat and is mediated by gut peptides, or satiety factors, released in response to nutritional stimuli. Cholecystokinin (CCK) is a satiety factor, released by enteroendocrine cells in the upper gastrointestinal tract in response to a meal. The release of CCK activates vagal afferents that project to the nucleus of the tractus solitaruis (NTS) and area postrema of the brainstem to initiate mechanisms that terminate a meal, such as a reduction in the rate of gastric emptying [28], [29]. The NTS also sends projections to nuclei within the forebrain that are involved in the regulation of food intake including the paraventricular nucleus (PVN) and arcuate nucleus of the hypothalamus [30]. The hypothalamus integrates peripheral information on short term energy intake via the gut. Exogenous (peripheral) administration of CCK in rodents reduces food intake by increasing satiation, and produces a characteristic pattern of neuronal activation, measured by the induction of c-Fos protein in the forebrain and brainstem including the supraoptic nucleus of the hypothalamus (SON), PVN, NTS and area postrema [31]–[34]. The NTS is also activated by endogenous satiety factors, including CCK, released by food ingestion as c-Fos protein expression is observed in this nucleus in response to food intake after a fast [35]–[37]. Furthermore, the degree of activation (measured by c-Fos expression) of the NTS is positively correlated to food intake that is presumably due to the postprandial release of endogenous satiety factors such as CCK [38]. It is possible, therefore, that the sensitivity of 3×TgAD mice to the satiety-inducing effects of food stimuli may be reduced, thus indicating a role for gut-brain signalling in the hyperphagia observed in these mice.

In the present study we characterised the feeding response in 3×TgAD mice and Non-Tg controls and tested the hypothesis that 3×TgAD mice have a reduced peripheral sensitivity to satiety signals. Specifically, we monitored the behavioural satiety sequence (BSS) in 3×TgAD mice and determined the anorexic responsiveness of these mice to CCK. The BSS is a well-characterised set of behaviours in response to a fast in rodents, including eating, followed by exploration, grooming and finally rest or sleep [39], [40]. Finally, as peripheral satiety signals act in the brainstem and hypothalamus via vagal stimulation, we determined changes in neuronal sensitivity by assessing c-Fos expression in the brains of 3×TgAD and NonTg mice following CCK administration and in response to food intake.

## Methods

### Ethics Statement

All experimental procedures using animals were conducted in accordance with the United Kingdom Animals (Scientific Procedures) Act, 1986 and approved by the Home Office and the local Animal Ethical Review Group, University of Manchester.

### Animals

3×TgAD and background strain, wild-type non-transgenic mice (Non-Tg) (C57BL6/129sv), were originally supplied by Frank LaFerla and Salvadore Oddo (University of California-Irvine, CA, USA) and in-house colonies were established. Male mice were group-housed in standard housing conditions (temperature 20±2°C, humidity 55±5%, 12 h light/12 h dark cycle with lights on at 07.00am), and given *ad libitum* access to standard rodent chow and water unless stated.

### Behavioural Satiety Sequence

3×TgAD and Non-Tg control mice (at 3 and 6 months of age; Non-Tg, *n* = 6–7, 3×TgAD, *n = *7–9) were housed individually in transparent observational cages and fasted overnight for 12 h. Water was available *ad libitum*. The following morning (3 h after beginning of the light phase, 10.00am), mice were given a pre-weighed amount of food and behaviour was monitored (see below). In a separate experiment, 3×TgAD and Non-Tg mice (at 12 months of age) were given an i.p. injection of CCK (8, 15, 30 µg/kg, *n = *7 per group; CCK 26–33, sulphated, Sigma-Aldrich Corp. Ltd., Poole, UK) or vehicle (saline, 5 ml/kg, *n = *7) after a 12 h fast. Following injections, mice were returned to their observation cages with a pre-weighed amount of food and behaviour was monitored.

For all mice, behaviour was recorded every 30 sec for 2 h (as reported previously [41]) and food intake was calculated. Behaviour was classified into: feeding (animal at hopper trying to obtain food, chewing, gnawing or holding food in paws), drinking (animal licking spout of water bottle), grooming (animal scratching, licking or biting any part of its anatomy), activity (including locomotion, sniffing, rearing), inactivity (immobility when aware, or signs of sickness behaviour), resting (animal curled up, resting head with eyes closed). Data were collated into 5-min period bins for display. Time spent in each of the behaviours as a % of the total behaviour, and the latency to rest (i.e. time at which animals first rested) were calculated.

### c-Fos Immunohistochemistry

3×TgAD and Non-Tg control mice (at 12 months of age) were individually housed a day prior to injections with *ad libitum* access to food and water. On the morning of the experiment mice were given an i.p. injection of either CCK (15 µg/kg, *n = *6) or vehicle (saline, 5 ml/kg, *n* = 6). The dose of CCK was based on results of the previous experiment due to its ability to decrease food intake in Non-Tg mice. In a separate experiment, groups of Non-Tg and 3×TgAD mice (*n* = 4–6/group) were either fasted for 12 h overnight or given *ad libitum* access to food. Mice were then either re-fed for 90 min, or remained fasted or fed *ad libitum* until sacrifice. Food intake was measured in the re-fed and *ad libitum* fed group at 90 min. All groups of mice were terminally anaesthetised with isoflurane (in 30% O_2_ and 70% nitrous oxide) 90 min after treatment or replacement of food, and transcardially-perfused with 0.9% heparinised saline followed by 4% paraformaldehyde (PFA, in 0.1 M phosphate buffer (PB)). Brains were removed and post-fixed overnight in 4% PFA followed by cryoprotection in 30% sucrose solution at 4°C, and then frozen. Coronal 30 µm brain sections were cut throughout the level of the hypothalamus and brainstem regions on a freezing sledge microtome and stored in cryoprotectant at −20°C until required. Immunohistochemistry for c-Fos protein was performed on free floating sections. After removal of endogenous peroxidase and treatment with blocking solution (2% normal goat serum in PB/0.3% triton), sections were incubated overnight at 4°C in a rabbit polyclonal anti-c-Fos antibody (1∶2000, Ab5, Oncogene Research Products, UK), washed in PB/0.3% triton, and incubated for 2 h in a peroxidase-labelled goat anti-rabbit IgG antibody (1∶500; Vector Laboratories Inc, UK). Following further washes (in 0.1 M PB), nuclear c-Fos was detected by incubation in a nickel-diaminobenzidine solution (Sigma-Aldrich, UK) that produced a blue-black precipitate. Sections were then mounted and coverslipped.

The number of immunopositive cells expressing c-Fos protein per section (2–12 sections depending on the region analysed) were counted bilaterally, using a light microscope, in nuclei defined by Paxinos and Franklin [42] as follows: supraoptic nucleus of the hypothalamus (SON): −0.58 to −0.94 mm; paraventricular nucleus of the hypothalamus (PVN): −0.58 to −1.22 mm; arcuate nucleus of the hypothalamus: −1.46 to −2.30 mm; nucleus of the tractus solitarius (NTS): −6.96 to −8.24 mm; area postrema: −7.20 to 7.76 mm. The average number of cells per section was then calculated, and the group mean determined for each brain region. The brain regions analysed for c-Fos protein expression were chosen as they are known to be involved in appetite regulation after fasting or re-feeding, or in response to i.p. injection of CCK in the mouse [31]–[33], [35]–[37].

### Statistical Analysis

All data are presented as mean ± the standard error of the mean (SEM) unless otherwise stated. Data for two groups was analysed by a Student's *t* test for parametric data and the Mann Whitney U test for non-parametric data. For more than two groups a one-way ANOVA or a two-way ANOVA followed by a Tukey's post hoc test was performed. Statistical significance was taken a *P*<0.05.

## Results

### Behavioural Satiety Sequence in 3×TgAD Mice

**Figure 1:**
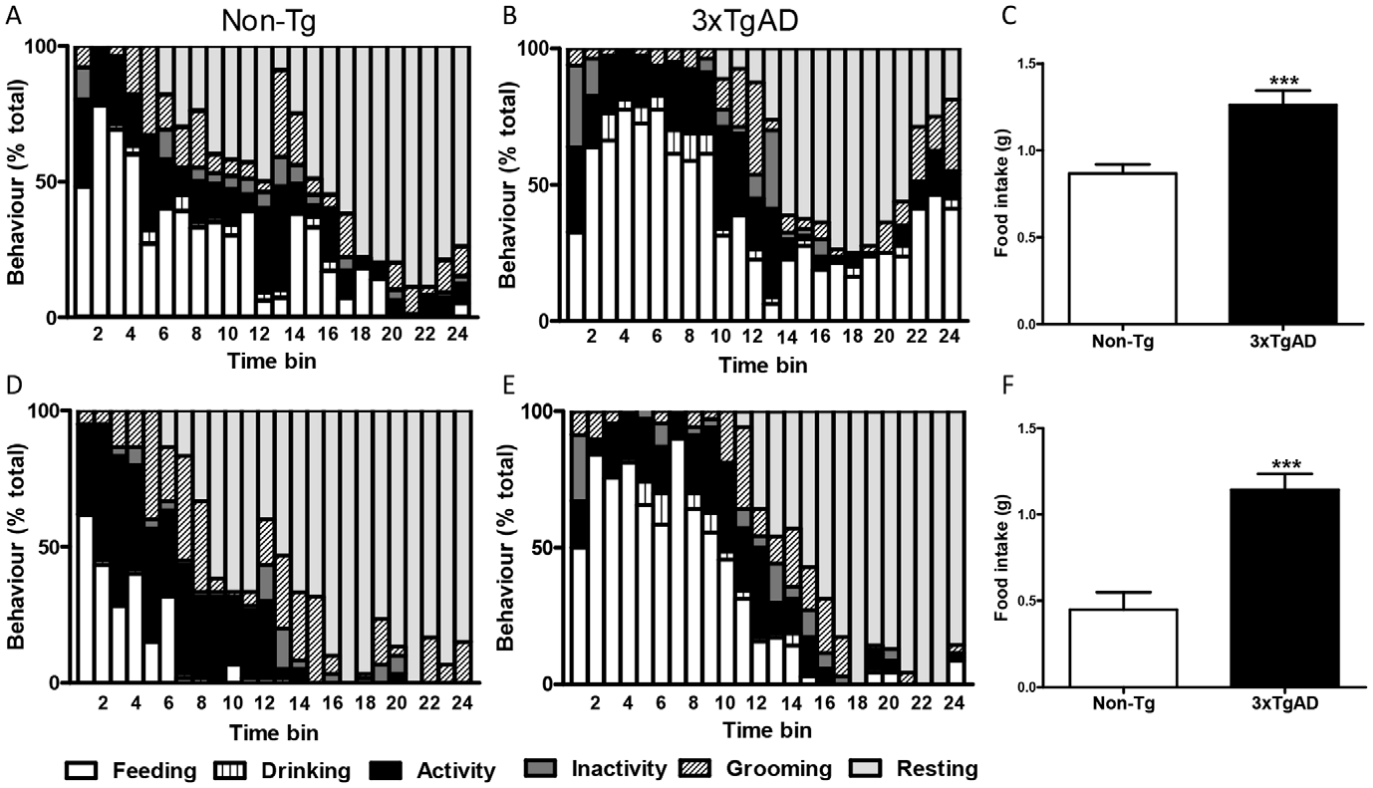
3×TgAD mice display increased feeding behaviour in the behavioural satiety sequence (BSS). Fasted (12 h) Non-Tg control (A and D) and 3×TgAD (B and E) mice at 3 (A–C) or 6 (D–F) months of age were presented with food and behaviour was monitored every 30 sec for 2 h and grouped into feeding, drinking, activity, inactivity, grooming or resting. Data were collated into 5-min time bins and are presented as percentage of total behaviour. Food intake (g) in 3- (C) and 6- (F) month-old Non-Tg and 3×TgAD mice was calculated at 2 h and is represented as mean ± SEM for n = 6–9 per group. ***P<0.001 versus Non-Tg controls, Student's *t* test. Statistical analyses of behaviours are presented in Table 1.

To assess if 3×TgAD mice displayed normal feeding behaviour the BSS was performed in 3- and 6- month-old mice. The BSS was maintained in 3×TgAD mice, as the overall pattern of behaviours was the same compared to age-matched Non-Tg controls (Figures 1A–B and D–E). Quantification of individual behaviours demonstrated that compared to Non-Tg controls 3×TgAD mice spent significantly more time feeding and drinking, and less time resting at both 3 and 6 months of age (*P*<0.05–0.001; Table 1). Grooming behaviour was also significantly decreased in 6-month-old 3×TgAD mice when compared to age matched controls (*P*<0.001). In both age groups, the average latency to rest was significantly longer in 3×TgAD versus Non-Tg control mice (*P*<0.05; Table 1). Food intake over 2 h was significantly greater in 3×TgAD mice compared to Non-Tg controls and was increased by 44% and 120% in 3- and 6-month-old mice respectively (*P*<0.001; Figures 1C and F). Body weight was significantly greater in 3×TgAD mice (3-month-old: Non-Tg, 21.9±0.5 g versus 3×TgAD, 28.2±0.5 g, *P*<0.001; 6-month-old: Non-Tg, 28.7±0.5 g versus 3×TgAD, 33.9±0.9 g, *P*<0.001, *n* = 6–9), however, when food intake was expressed as g/kg body weight there was still a significant increase in food consumed in 6-month-old 3×TgAD mice (3-month-old: Non-Tg, 39.6±2.5 g/kg versus 3×TgAD, 44.8±2.9 g/kg, *P*>0.05; 6-month-old: Non-Tg, 15.7±3.5 g/kg versus 3×TgAD, 33.7±2.7 g, *P*<0.01).

**Table 1:** The behavioural satiety sequence (BSS) in 3×TgAD and Non-Tg control mice.

	3 months		6 months	
Behaviour (% time)	Non-Tg	3×TgAD	Non-Tg	3×TgAD
Feeding	26.7±2.3	40.7±5.4*	9.7±2.1	32.1±3.1***
Drinking	1.8±0.2	3.2±0.7*	0.3±0.2	2.2±0.4**
Activity	14.3±1.3	15.1±1.6	18.4±3.8	12.5±2.4
Inactivity	3.7±0.7	4.8±1.5	3.1±0.8	4.2±0.5
Grooming	10.8±1.1	8.7±1.4	14.4±0.8	7.8±0.6***
Resting	39.9±2.0	27.5±5.0*	54.3±4.8	41.4±3.9*
Latency to rest (min)	44.0±4.7	67.5±8.2*	46.7±8.4	67.9±4.6*

3×TgAD and Non-Tg control mice were fasted for 12 h, food was then returned and behaviour monitored for 2 h. The percentage of time spent in each behaviour was calculated and is represented as mean ± SEM for n = 6–9 per group.

* *p*<0.05,

** *p*<0.01,

*** *p*<0.001 versus Non-Tg controls, Student's *t* test. Graphical representation of these data is presented in Figure 1.

### The effect of CCK on Food Intake and the BSS in 3×TgAD Mice

**Figure 2:**
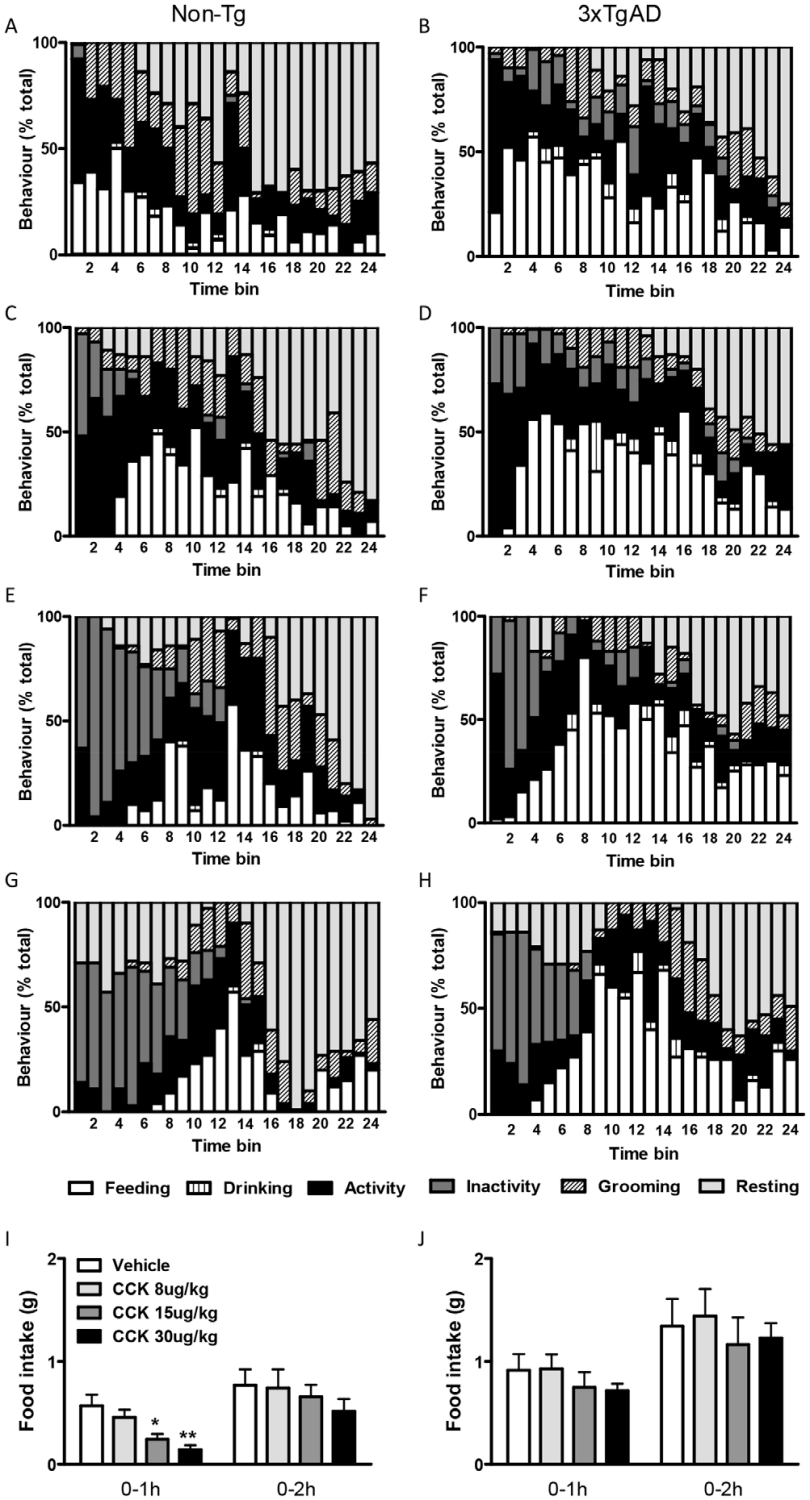
The effect of cholecystokinin (CCK) on the behavioural satiety sequence (BSS) in 3×TgAD and Non-Tg control mice. Non-Tg control (A, C, E, G and I) or 3×TgAD (B, D, F, H and J) mice were fasted (12 h) and given an i.p injection of vehicle (saline 0.9% NaCl, A–B) or CCK at 8 µg/kg (C–D), 15 µg/kg (E–F) or 30 µg/kg (G–H). Behaviour was subsequently monitored at 30-sec intervals for 2 h and grouped into feeding, drinking, activity, inactivity, grooming or resting. Data were collated into 5-min time bins and are presented as percentage of total behaviour. Food intake (g) in Non-Tg (I) and 3×TgAD (J) mice after CCK injection was calculated at 1 and 2 h and is represented as mean ± SEM for n = 7 per group. * *P*<0.05, **P<0.01 versus vehicle-treated mice. Statistical analyses of behaviours are presented in Table 2.

CCK dose-dependently reduced food intake in control Non-Tg mice 1 h after i.p. injection with no difference being observed at 2 h (Figure 2I). In contrast, CCK had no affect on food intake in 3×TgAD mice at 1 or 2 h post injection at any dose tested (Figure 2J). Vehicle-treated Non-Tg and 3×TgAD mice showed a normal BSS over 2 h (Figures 2A and B). All doses of CCK appeared to partially disrupt the BSS in Non-Tg mice and a dose-dependent increase in a period of inactivity was observed immediately after injection that lasted up to 1 h (Figures 2A, C, E and G). When individual behaviours were assessed, 15 and 30 µg/kg CCK significantly increased the % of time Non-Tg mice were inactive compared to vehicle-treated Non-Tg mice (*P*<0.01 and *P*<0.05 respectively, Table 2). In addition, 30 µg/kg CCK also decreased the amount of time spent grooming in Non-Tg mice (*P*<0.05). However, although i.p. injection of CCK appeared to slightly disrupt the BSS in 3×TgAD mice, with a dose-dependent increase in inactive behaviour being observed immediately after injection (Figures 2B, D, F and H), there was no significant difference in the % of time spent in any of the behaviours analysed (Table 2). CCK had no affect on the latency to rest in Non-Tg or 3×TgAD mice.

**Table 2:** The effect of cholecystokinin (CCK) on the behavioural satiety sequence (BSS) in 3×TgAD and Non-Tg control mice.

	Non-Tg				3×TgAD			
Behaviour (% time)	Vehicle	CCK 8 µg/kg	CCK 15 µg/kg	CCK 20 µg/kg	Vehicle	CCK 8 µg/kg	CCK 15 µg/kg	CCK 20 µg/kg
Feeding	18.3±4.1	22.2±6.7	15.0±2.3	14.0±3.3	32.5±8.7	34.6±9.0	35.1±8.0	29.0±4.9
Drinking	1.1±0.5	1.1±0.4	0.7±0.2	0.4±0.2	2.5±1.1	3.2±0.9	2.5±1.1	2.1±0.6
Activity	21.7±2.8	27.5±2.7	23.9±2.9	13.9±3.1	23.8±2.6	27.0±5.6	23.8±2.2	19.1±3.4
Inactivity	0.6±0.5	6.2±2.6	21.1±3.5**	20.2±5.8*	8.8±4.0	10.0±5.6	12.0±3.2	14.7±4.7
Grooming	19.6±3.2	15.1±2.1	13.1±2.2	9.8±1.5*	11.3±2.9	8.5±1.8	7.3±2.0	10.0±1.5
Resting	39.6±5.6	28.9±6.2	26.3±5.2	41.7±6.9	21.1±6.5	16.7±6.9	19.3±7.5	25.1±7.9
Latency to rest (min)	44.3±4.8	44.3±4.8	60.0±14.3	53.6±12.7	80.0±12.5	97.1±9.0	76.7±15.4	70.7±17.1

3×TgAD and Non-Tg control mice were fasted for 12 h and given an i.p. injection of CCK (8, 15, or 30 µg/kg) or vehicle (saline). Food was then returned and behaviour monitored for 2 h. The percentage of time spent in each behaviour was calculated and is represented as mean ± SEM for n = 7 per group.

* *p*<0.05,

** *p*<0.01 versus vehicle-treated mice. Graphical representation for these data is presented in Figure 2.

### The effect of CCK on c-Fos Expression in the 3×TgAD Mouse Brain

**Figure 3:**
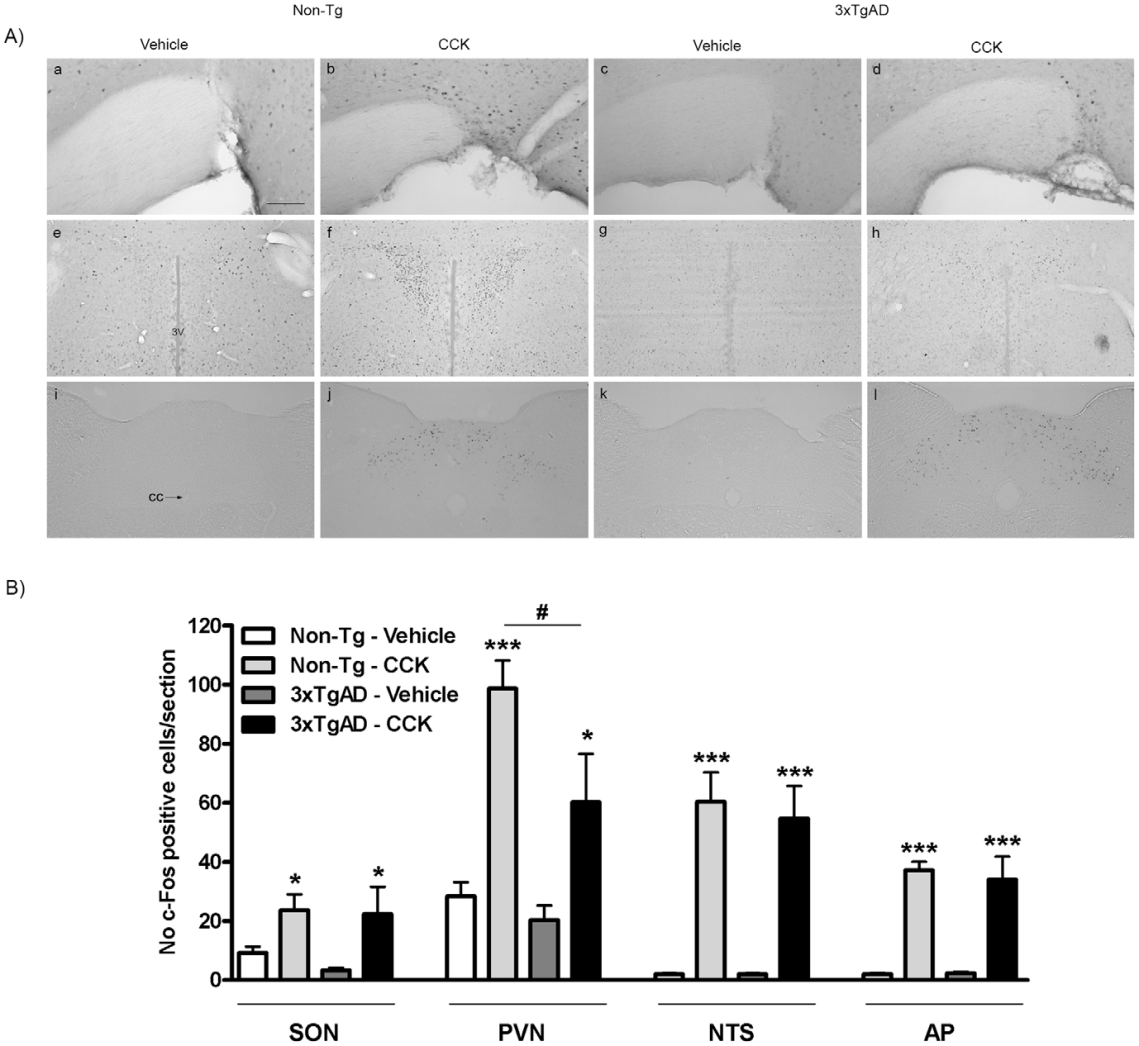
The effect of cholecystokinin (CCK) on c-Fos expression in the brain of 3×TgAD and Non-Tg control mice. (A) Representative photomicrographs illustrating c-Fos expression in the brain of Non-Tg control (a, b, e, f, i, j) or 3×TgAD (c, d, g, h, k, l) mice after i.p. injection of either vehicle (5 ml/kg saline, *n* = 6) or CCK (15 µg/kg, *n* = 6). Significant increases in c-Fos expression were observed after CCK injection in the supraoptic nucleus of the hypothalamus (SON; a–d), paraventricular nucleus of the hypothalamus (PVN; e–h), nucleus tractus solitarius (NTS; i–l) and area postrema (AP; i–l) of Non-Tg control and 3×TgAD mice, although the number of c-Fos-positive cells in response to CCK was lower in PVN of 3×TgAD compared to Non-Tg control mice. Scale bar, 50 µm for a–d and 100 µm for e–l. 3V, third ventricle; cc, central canal. (B) quantification of the number of c-Fos positive nuclei per section. Data are mean ± SEM, *P<0.05, ***P<0.001 versus respective vehicle-treated mice, ^#^ *P*<0.05 versus CCK-treated Non-Tg mice, Two-way ANOVA.

I.p. injection of CCK (15 µg/kg) caused a significant increase in c-Fos protein expression in the SON, PVN, NTS and AP of brains from both Non-Tg and 3×TgAD mice compared to respective vehicle-treated controls (*P*<0.05 and *P*<0.001; Figure 3). The effect of CCK in 3×TgAD mice was of a similar magnitude to Non-Tg mice, as no difference was detected in the number of c-Fos-positive cells between CCK-treated 3×TgAD and Non-Tg mice in the SON, NTS and AP. However, the number of c-Fos positive cells in the PVN of CCK-treated 3×TgAD mice was significantly less than in Non-Tg mice injected with CCK. No difference in c-Fos expression was observed in vehicle-treated 3×TgAD compared to Non-Tg mice in all nuclei examined.

### The effect of a Fast and Fast-Induced Feeding on c-Fos Expression in the 3×TgAD Mouse Brain

In order to compare the responsiveness of 3×TgAD mice to endogenous satiety factors, the level of c-Fos expression was compared in the brains of Non-Tg and 3×TgAD mice that were either fed *ad libitum*, fasted, or allowed to re-feed for 90 min after a fast. 3×TgAD mice ate significantly more food during the 90 min re-feed after the 12 h fast compared to Non-Tg mice (Non-Tg, 0.8±0.2 g versus 3×TgAD, 1.8±0.2 g; *P*<0.01). No food was consumed during the 90 min monitoring period in *ad libitum* fed Non-Tg or 3×TgAD mice.

**Figure 4:**
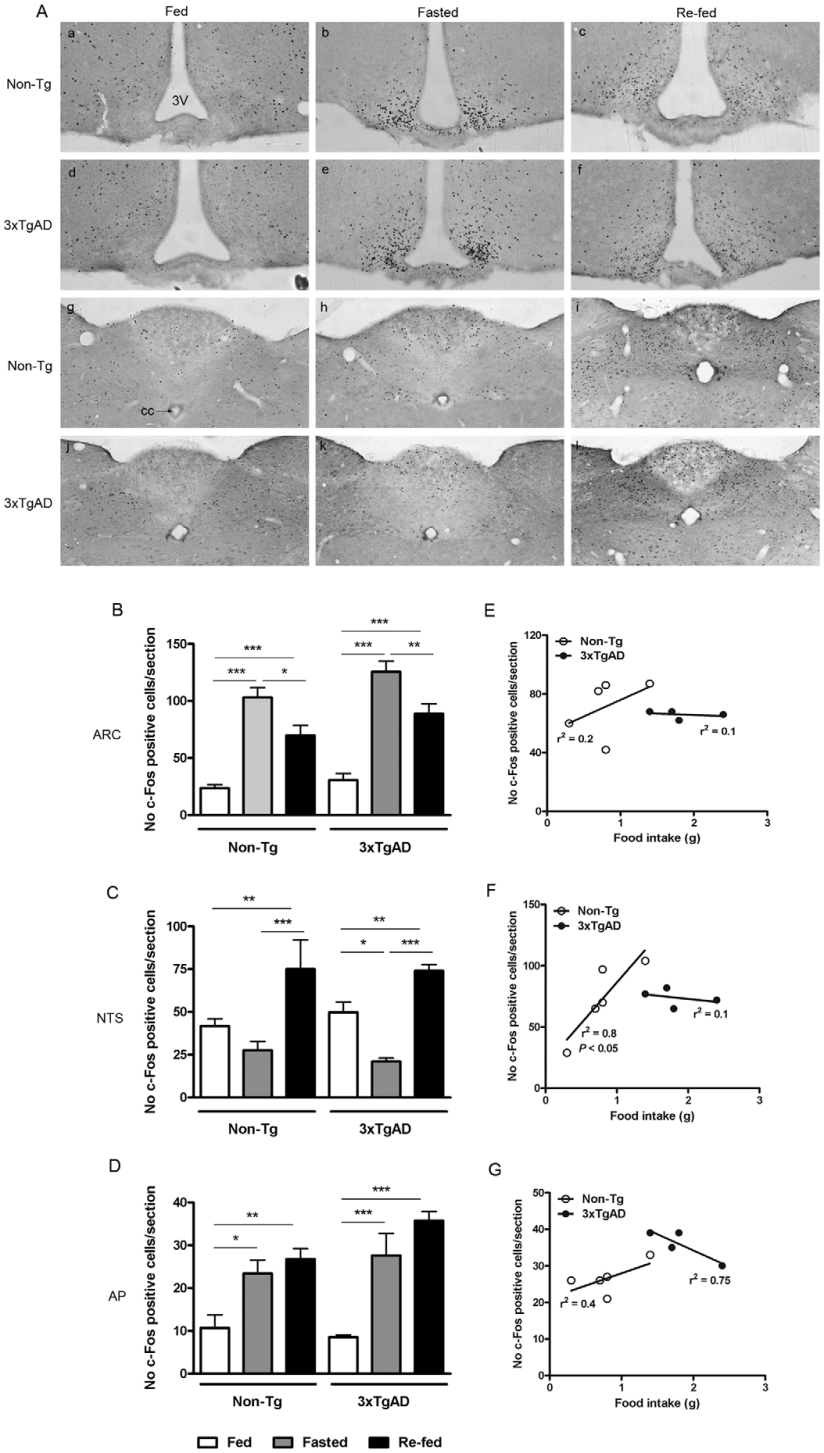
The effect of fasting and re-feeding on c-Fos expression in the brain of 3×TgAD and Non-Tg mice. (A) Representative photomicrographs illustrating c-Fos expression in the arcuate nucleus of the hypothalamus (ARC) (a–f), nucleus tractus solitaries (NTS) (g–l) and area postrema (AP) (g–l) of *ad libitum* fed, 12 h fasted, or 90 min re-fed Non-Tg (a–c, g–i) or 3×TgAD (d–f, j–l) mice (*n* = 4–6/group). Scale bar, 100 µm. 3V, third ventricle; cc, central canal. Quantification of the number of c-Fos positive nuclei per section are represented in (B) for the ARC, (C) for the NTS and (D) for the AP. Data are mean ± SEM, *P<0.05, **P<0.01, ***P<0.001, Two-way ANOVA. (E–G) Linear regression analysis indicates that the number of c-Fos positive cells in the NTS (F) of Non-Tg mice correlates positively with food intake (*P*<0.05) but there is no relationship between food intake and c-Fos expression in 3×TgAD mice. No correlation between food intake and the number of c-Fos positive cells were detected in the ARC (E) or AP (G) of Non-Tg or 3×TgAD mice.

Fasting significantly increased c-Fos expression in the arcuate nucleus of the hypothalamus in both Non-Tg and 3×Tg-AD mice compared to their respective fed controls (*P*<0.001; Figures 4A and B). Re-feeding decreased the number of c-Fos positive neurones in the arcuate nucleus of Non-Tg and 3×TgAD mice when compared to the fasted state (*P*<0.05 and *P*<0.01 respectively) but a significant increase was still detected compared to fed controls in both Non-Tg and 3×TgAD mice (*P*<0.001).

In the NTS (Figure 4A and C) of Non-Tg mice, fasting had no effect on c-Fos expression compared to fed controls but 90 min re-feeding caused a significant increase in the number of c-Fos positive cells compared to the fed and fasted state (*P*<0.01 and *P*<0.001 respectively). In contrast, fasting reduced c-Fos expression in the NTS of 3×TgAD mice (*P*<0.05) and re-feeding reversed this effect with a significant increase in the number of c-Fos profiles observed in the NTS of re-fed 3×TgAD compared to fed or fasted 3×TgAD mice (*P*<0.01 and *P*<0.001 respectively).

Fasting increased the number of c-Fos positive cells in the AP of both Non-Tg and 3×TgAD mice (*P*<0.05 and *P*<0.001 respectively). Re-feeding had no effect on the degree of c-Fos expression in the AP when compared to fasting in both 3×Tg-AD and Non-Tg mice but a significant increase in c-Fos positive cells was observed after re-feeding compared to fed controls in both mice (*P*<0.01 and *P*<0.001; Figure 4A and C).

In Non-Tg mice, the degree of activation of the NTS after a re-feed was related to food intake as linear regression analysis revealed that the number of c-Fos positive cells in the NTS was positively correlated to the amount of food consumed (r^2^ = 0.8, *P<*0.05, Figure 4F). However, there was no correlation between food intake and c-Fos expression in the NTS of 3×TgAD mice in response to a re-feed (r^2^ = 0.1, *P*>0.05, Figure 4F). No correlation between food intake and c-Fos expression was observed in the arcuate nucleus of the hypothalamus (Figure 4E) or the area postrema (Figure 4G) in Non-Tg or 3×TgAD mice.

## Discussion

AD is associated with several non-cognitive changes in behaviour such as weight loss and alterations in eating patterns. In spite of the weight loss observed in patients with AD, appetite is often increased and calorie intake is adequate for the body's requirements [13], [17]–[19], [22], [43], although in severe and late-stage AD patients usually present with difficulties in eating and malnutrition [44]–[46]. Increased appetite has been reported in some AD mouse models including 3×TgAD mice [25]–[27]. We have demonstrated previously that at 2 months of age 3×TgAD mice are hyperphagic and have higher body mass. Increased food intake is still present in 12-month-old 3×TgAD mice but at this age these mice weigh less than Non-Tg controls, an effect that is likely to be due to an increase in metabolic rate [25]. The present study sought to determine the mechanisms underlying the enhanced appetite in 3×TgAD mice.

Detailed analysis of the BSS revealed that the overall pattern of the behavioural response to fast-induced food intake in 3- and 6-month-old 3×TgAD mice was normal, and an increase in food intake in 3×TgAD mice was accompanied by enhanced feeding and drinking behaviour. Consistent with hyperphagia, the BSS in 3×TgAD mice was shifted to the right as indicated by an increase in the latency to rest and less time spent in rest over the monitoring period. As the BSS monitors the natural sequence of behaviours in rodents in response to food intake, and therefore reflects physiological satiety [40], the current study indicates that 3×TgAD mice have greater appetites and do not exhibit abnormal ingestive behaviours. Increased appetite could be a consequence of higher body mass, as 3×TgAD mice weigh more than controls at 3 and 6 months of age as shown here and previously (e.g. [25], [47]). However, food intake is still greater in 6-month-old 3×TgAD mice when food intake is expressed as g/kg body weight.

Mechanisms responsible for the changes in appetite in AD are unknown. Appetite is regulated by short and long term mediators, including the anorexic factors CCK and leptin respectively (for recent reviews see [48]–[50]). Ingestion of food, and the subsequent release of nutrients (e.g. lipids and proteins), activates enteroendocrine cells of the gastrointestinal tract to secrete anorexigenic peptides such as CCK and peptide YY (PYY). These peptides mediate their actions by entering the circulation, giving them access to the brain via circumventricular organs, or by activation of vagal afferents that project from the stomach to the dorsal vagal complex (DVC) in the brainstem. The DVC includes the NTS, area postrema and the dorsal motor nucleus of the vagus. Activation of the DVC also signals to areas of the hypothalamus, such as the arcuate nucleus and the PVN, both of which have been implicated in the regulation of food intake. Vagal efferent nerve fibres run from the dorsal motor nucleus of the vagus back to the gastrointestinal tract, feeding back information concerning energy homeostasis and food intake. These vagal efferent nerves innervate the stomach and reduce gastric emptying, which induces satiety. Peripheral administration of CCK causes a dose dependent reduction in food intake in rodents and increases c-Fos expression (a marker of cell activation) in the NTS and area postrema of the brainstem, and the SON and PVN of the hypothalamus [31]–[34]. The present study also demonstrated that exogenous administration of CCK caused a dose-dependent reduction in food intake in Non-Tg control mice. In addition, CCK partially disrupted the BSS in Non-Tg mice, as a period of inactivity was observed immediately after injection. This increase in inactive behaviour was probably due to sickness behaviour and conditioned taste aversion that CCK can induce at high doses [51]–[54]. However, in 3×TgAD mice CCK had no significant affect on food intake or behaviour in the BSS. These data therefore suggest that 3×TgAD mice were insensitive to the anorectic actions of peripheral administration of CCK. The insensitivity of 3×TgAD mice to exogenous CCK was unlikely to be due to impaired vagally-mediated signalling in the brainstem as CCK increased c-Fos to the same degree in the NTS and AP of 3×TgAD when compared to Non-Tg control mice. However, less c-Fos positive cells were observed in the PVN of the forebrain of CCK-treated 3×TgAD mice. Together these data suggest that the integration of signalling regarding nutritional status from the NTS to the hypothalamus may be impaired in 3×TgAD mice. Furthermore, deficits in efferent signalling from the DVC to the stomach may also be impaired and/or other downstream mediators of satiety may be affected in 3×TgAD mice. Alternatively, as 3×TgAD mice eat more, it is possible that a higher dose of CCK is needed to affect food intake due to the greater orexigenic drive in these mice. However, the dose of CCK used in the present study is likely to be supraphysiological and is therefore not a true reflection of natural satiety in response to a meal. Thus, we also tested the sensitivity of 3×TgAD mice to endogenous satiety factors by examining c-Fos expression in the brain in response to food ingestion. There was no difference in the level of c-Fos expression in the brainstem or forebrain after re-feeding between 3×TgAD and Non-Tg mice when compared to a fast indicating that key brain regions involved in appetite control are stimulated to the same degree in 3×TgAD and Non-Tg mice. However, as the degree of c-Fos expression in the NTS in response to re-feeding is dependent on food intake [38], a greater level of c-Fos positive cells should be observed in 3×TgAD mice as these mice ate more during the re-feed period. As previously reported [38] there was a positive correlation between food intake and Fos in the NTS of re-fed Non-Tg controls but this relationship was not observed in 3×TgAD mice. It is possible therefore, that in 3×TgAD mice, less CCK (or other satiety factors) are released in response to a meal. In support, meal-induced increases in CCK are reduced in obese rats that are also less sensitive to the anorexic effects of this satiety factor [55]–[57]. In summary, these data therefore suggest that 3×TgAD mice release a reduced amount of, and/or are less sensitive to, endogenous satiety factors in response to food ingestion possibly due to impairments in gut-brainstem signalling. In order to study this further, c-Fos expression in the NTS could be examined in 3×TgAD mice there were restricted to consume the same amount of food as Non-Tg control mice in response to a fast.

In addition to satiety factors (anorexic peptides/hormones), food intake is also regulated by orexigenic peptides/hormones that increase appetite. During periods of low energy intake or during fasting, the release of orexigenic peptides inform the brain of the nutritional status and produce metabolic, hormonal and behavioural changes to conserve energy and find an energy source [50], [58]. Increased food intake in 3×Tg-AD mice could therefore be caused by an increase in orexigenic peptide signalling. In rodents fasting induces c-Fos expression in several key brain nuclei involved in energy balance including the arcuate nucleus of the hypothalamus and the area postrema [59], [60]. In particular food deprivation robustly activates neurones containing the orexigenic peptide neuropeptide Y (NPY) in the arcuate nucleus of the hypothalamus [61], [62], an effect that is reversed by refeeding [63], [64]. In agreement, in the present study, fasting induced an increase in c-Fos expression in the arcuate nucleus and area postrema in Non-Tg control mice but also to the same degree in 3×TgAD mice. However, in response to fasting 3×TgAD mice had less c-Fos positive cells in the NTS when compared to fed mice but no difference between the fed and fasted state were observed in control Non-Tg mice. Fasting has been reported previously to have no affect on c-Fos expression in the NTS of rats (e.g. [35], [65]). Thus, the significance of the decreased c-Fos expression in the NTS of fasted 3×TgAD mice is unclear.

Few studies have examined the mechanisms for hyperphagia and altered appetite in AD patients. To date, studies have focussed mainly on potential neurochemical changes within the AD brain and have demonstrated a reduction in the density of receptors for the anorexic neuropeptide serotonin (5HT) [66]. Furthermore, alterations in the 5HT system appear to correlate with changes in eating behaviour as a reduction in 5HT-4 receptor is present in hyperphagic but not normophagic AD patients [67]. However, the data in the present study suggests that AD patients are likely to have impairments in the physiological control of appetite including the mechanisms controlling the termination of a meal (satiation). In support, hyperphagic AD patients have marked reductions in satiation when compared to non-hyperphagic AD subjects [20].

In summary, the 3×TgAD mouse model of AD shows increased food intake that is accompanied by a reduction in the sensitivity to the anorexic actions of exogenous and endogenous satiety factors. This decrease in the responsiveness of 3×TgAD mice to satiety might be meditated by a deficit in how the brain responds to endogenous satiety factors released in response to food ingestion. These data might help in understanding the hyperphagia that is reported in some AD patients.
